# Tailgut Cyst and Perineal Hydatid Cyst: A Case Report with Multimodality Imaging Findings

**DOI:** 10.1155/2016/4212753

**Published:** 2016-08-17

**Authors:** Ibtisam Musallam Aljohani, Khalefa Ali Alghofaily, Sebastian R. McWilliams, Mnahi Bin Saeedan

**Affiliations:** ^1^Ministry of National Guard, Health Affairs, King Abdulaziz Medical City, Medical Imaging Department, Riyadh, Saudi Arabia; ^2^College of Medicine, Medical Imaging Department, Al-Qassim University, Buraydah, Saudi Arabia; ^3^Mallinckrodt Institute of Radiology, Washington University, St. Louis, MO, USA; ^4^King Faisal Specialist Hospital & Research Center, Department of Radiology, P.O. Box 3354, Riyadh 1121, Saudi Arabia

## Abstract

A tailgut cyst is a rare developmental lesion and usually is located in the retrorectal or presacral space. Extrahepatic hydatid disease has been reported in several locations including the pelvis and it often poses a diagnostic challenge. There are very few reported cases of primary perineal hydatid cysts. We present the multimodality imaging findings of a tailgut cyst and concurrent perineal hydatid disease in a 32-year-old male patient.

## 1. Introduction

A tailgut cyst is a rare congenital lesion. It has a female predilection and is usually asymptomatic and detected during middle age [1]. It is usually located in the presacral space and seen as a discrete and well-defined mass of variable attenuation on computed tomography (CT) depending on its contents. Cyst wall calcification may be seen. Rarely, a tailgut cyst may be complicated by infection or malignant transformation [2–4].

Hydatid disease is a zoonotic parasitic disease.* Echinococcus granulosus* and* Echinococcus multilocularis* are the two main infectious causative agents. Although liver and lung are the most commonly affected organs, hydatid cysts have the potential to affect any part of the human body. Primary extrahepatic hydatid cysts have been reported in different organs and anatomical locations and generally pose a diagnostic challenge [5, 6]. There are few reported cases of hydatid disease involving the perineal region [7–9]. We present the multimodality imaging findings of a retrorectal/presacral tailgut cyst with concomitant hydatid disease of the perineum.

## 2. Case Report

**Figure 1:**
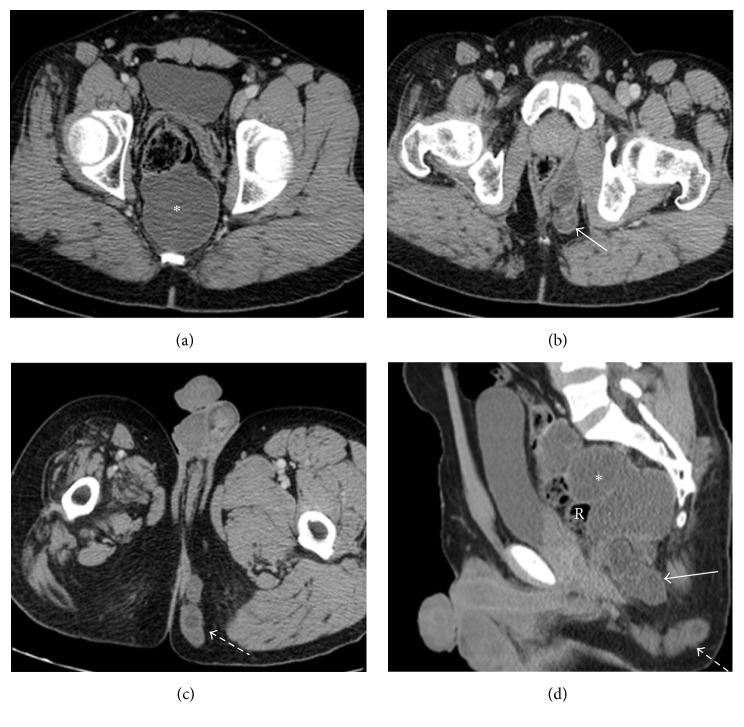
Multiple axial and sagittal contrast enhanced CT scan show a multilocular, hypoattenuating, retrorectal cystic lesion (asterisks) displacing the rectum (R) anteriorly. The left ischioanal fossa cystic lesion (solid arrow) shows enhancing wall and internal septations, breaching the left levator ani muscle and extending into the left perineal subcutaneous fat (dashed arrow). Atrophy of the right hemipelvis and proximal thigh muscles is noted and related to known poliomyelitis.

**Figure 2:**
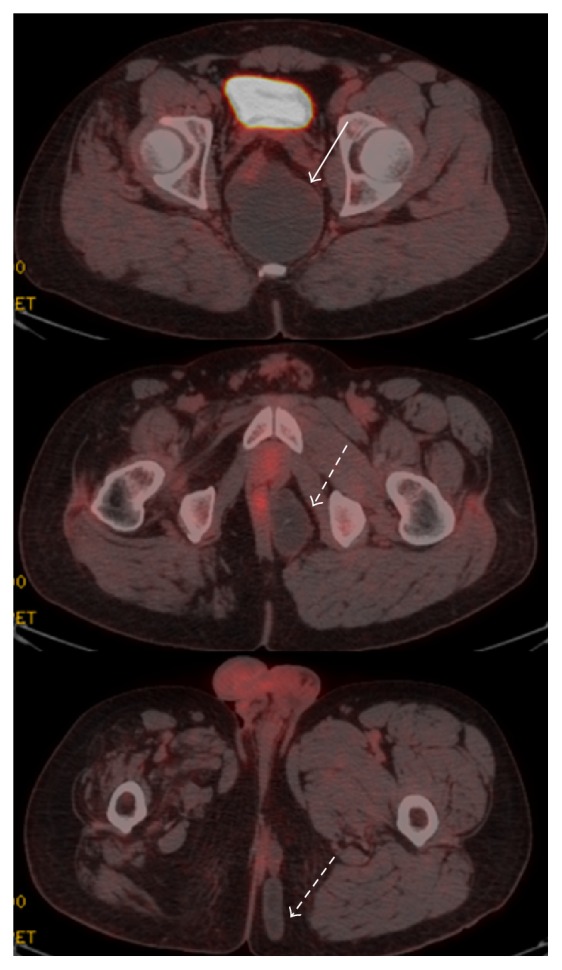
Fused FDG PET/CT images show no FDG uptake in the known retrorectal tailgut cyst (solid arrow) or the left ischioanal and perineal subcutaneous hydatid cysts (dashed arrows).

A 32-year-old male patient with a history of poliomyelitis in childhood complained of a progressively enlarging painless deep perianal mass for 1 year with a history of intermittent chronic constipation. The patient was referred to a tertiary healthcare center as a possible case of retroperitoneal sarcoma. Routine contrast enhanced CT showed a large (7.2 × 7.9 × 15.5 cm) multilocular, hypoattenuating, retrorectal cystic lesion. It displaced the rectum and rectosigmoid anteriorly and had no relation with the prostate, seminal vesicles, or adjacent major vessels. The CT also revealed a collection of smaller contiguous cysts in the left ischioanal fossa and the left perineal subcutaneous fat. This collection of cysts paralleled and was adjacent to the left puborectalis muscle and breached the left levator ani. No other lesion was found in the abdominal cavity. Atrophy of the right hemipelvis and proximal thigh muscles was related to the history of poliomyelitis (Figure 1). An ^18^F-fluorodeoxyglucose (FDG) positron emission tomography (PET/CT) scan was performed and showed no abnormal uptake in these lesions; no regional lymphadenopathy or distant metastases were seen (Figure 2).

**Figure 3:**
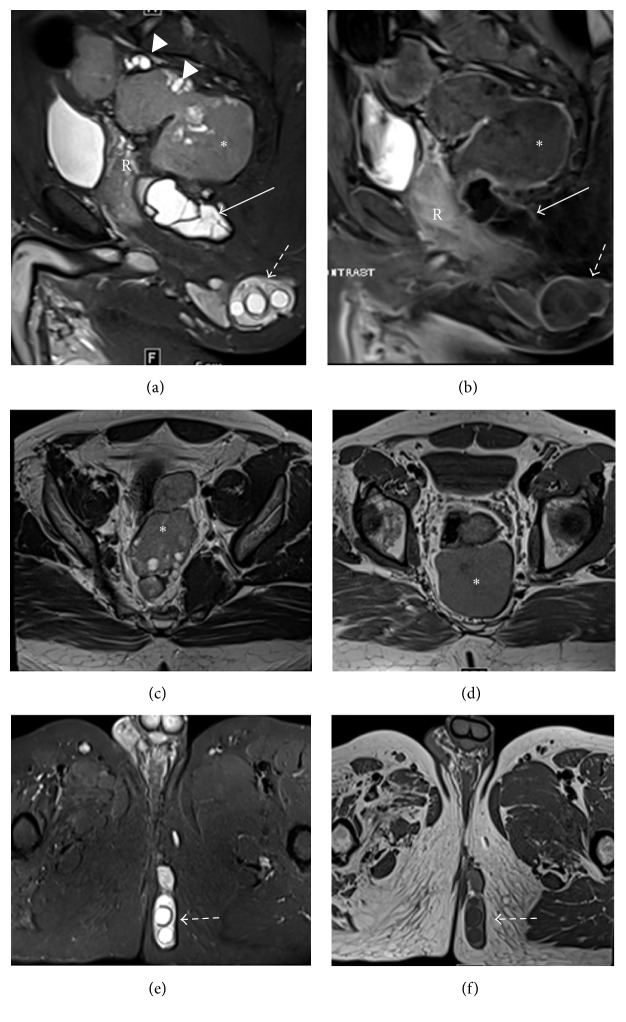
(a) Sagittal T2-weighted MR image with fat saturation shows a predominantly intermediate T2 signal intensity presacral/retrorectal multilocular lesion (asterisk) with multiple internal T2 bright foci and smaller peripheral cysts (arrowheads). Note the anterior displacement of the rectum. The left ischioanal (solid arrow) and subcutaneous perineal hydatid cysts (dashed arrow) show high T2 signal intensity with dark internal septations and rims. (b) Sagittal T1-weighted postcontrast MR image shows peripheral rim enhancement of the retrorectal tailgut cyst (asterisks) as well as the ischioanal (solid arrow) and perineal subcutaneous hydatid cysts (dashed arrow). ((c) and (d)) Axial T2-weighted (c) and T1-weighted (d) MR images show the retrorectal lesion of intermediate signal intensity (asterisk) with peripheral small cyst. ((e) and (f)) Axial T2-weighted (e) and T1-weighted (f) MR images show the left subcutaneous perineal hydatid cysts (dashed arrow) of high T2 signal intensity and intermediately low T1 signal intensity with internal daughter cysts and a dark rim.

Pelvic magnetic resonance imaging (MRI) was performed. The retrorectal mass showed intermediate signal intensity on both T1 and T2 with multiple internal T1 hypointense foci and T2 hyperintense foci. The left ischioanal fossa collection of cysts showed low T1 and high T2 signal intensity with multiple internal cysts and T2 and T1 hypointense rim. There was thin peripheral enhancement with no enhancing mural solid component. There was no osseous involvement (Figure 3).

The patient underwent surgical resection. The histopathologic examination of the resected retrorectal cyst revealed a benign tailgut cyst. The resected left ischioanal and gluteal cystic lesions showed an outer histiocytic reaction to a hydatid cyst.

## 3. Discussion

The unique feature of this primary extrahepatic hydatid cyst is its very unusual location in the ischioanal fossa with extension to the left perianal and gluteal areas. While the hydatid cysts had increased signal on T2-weighted images, the tailgut cyst had intermediate signal, denoting proteinaceous or mucinous content. The presence of both a perineal hydatid cyst and tailgut cyst with differing signal characteristics on MRI in a contiguous location in one patient imposed a diagnostic challenge where the added information of MRI proved very useful. In fact, the interpreting radiologist suggested a developmental lesion such as a tailgut cyst and atypical hydatid disease. The differential diagnosis might include a myxoid tumor such as myxoid neurofibroma or aggressive angiomyxoma due to the presence of myxoid, proteinaceous, or mucinous contents. However, the lack of an enhancing solid component makes those diagnoses less likely.

A tailgut cyst is a rare developmental lesion that is a remnant of the embryonic tailgut. The tailgut is the most caudal part of the hindgut that normally involutes during embryonic development. Tailgut cysts have a female predilection, are usually asymptomatic, and are often detected in middle age. They can be discovered at any age and may cause symptoms such as abdominal pain or constipation [1].

The imaging findings of tailgut cysts on CT and MRI have been described. Usually, CT shows a presacral, discrete, and well-marginated lesion of fluid or soft tissue attenuation depending on the cyst contents. Cyst wall calcifications may be seen and the rectum may be displaced if the lesion is large [2–4]. On MRI, tailgut cysts are usually hyperintense on T2-weighted images and hypointense on T1-weighted images. However, the presence of high protein content, mucinous materials, or internal hemorrhage may lead to high T1 signal intensity [4, 10, 11]. Malignant transformation of the tailgut cyst has been reported. Loss of discrete margins and involvement of contiguous structures can be detected on CT and might suggest the cyst is complicated by infection or malignant transformation [2]. Fibrous tissue within the cyst or malignant transformation may be seen as enhancing irregular wall thickening or a polypoid mass of intermediate signal intensity on both T1- and T2-weighted images [11, 12].

Because of the malignant potential of a tailgut cyst, to distinguish it from the other presacral cysts is essential. Differential diagnoses of a presacral cystic lesion might include rectal duplication cyst, dermoid cyst, epidermoid cyst, cystic lymphangioma, and anterior meningocele. These diagnoses are usually unilocular except for a tailgut cyst and cystic lymphangioma which are usually multicystic [13, 14]. Tailgut cysts have been reported as unilocular or multilocular cystic lesions on MRI [4, 10, 11]. Some suggest that a T2-weighted multilocular appearance with internal septa is a unique feature of a tailgut cyst [10, 14]. Another study observed the presence of small peripheral cysts within a large cyst [14]. Therefore, MRI and specifically T2-weighted MR images offer a better depiction of the multilocular nature and detection of any smaller internal cysts. As compared to CT, MRI offers better tissue contrast. Sagittal MRI can be used to assess the relationship of the presacral lesion with the rectum and osseous structures. Nevertheless, there is a considerable overlap in the imaging findings of presacral cystic lesions, and therefore histologic evaluation is essential to the establishment of a definitive diagnosis of tailgut cyst [10, 14].

*Echinococcus granulosus* and* Echinococcus multilocularis *are the two main infectious causative agents of hydatid disease. Dogs are usually the definitive hosts and sheep are the most common intermediate hosts. Humans are an intermediate host and may become infected after ingestion of food contaminated with* Echinococcus* eggs. Hydatid cysts have three components, the pericyst, middle laminated membrane, and the inner germinal layer. The pericyst is the surrounding host tissue inflammatory response. The scolex (the infectious parasite at the larval stage) arises from the inner endocyst [5, 6]. Although not highly sensitive or specific, serologic tests for the detection of* Echinococcus* antibody should be part of the work-up of any detected cystic lesion in an unusual location.

In humans, hydatid disease affects the liver, the lung, and other locations in approximately 75%, 15%, and 10% of cases, respectively [5, 6]. When occurring in unusual locations, most cases are secondary hydatid disease. Primary hydatid disease is considered when no other cysts are detected in the more common sites of occurrence. Primary pelvic hydatid disease has been reported with an incidence of 2%. It has be postulated that the hydatid scolex gains access to the pelvis by the lymphatic or haematogenous route [15, 16]. There are three reported cases of hydatid disease involving the perineal region [7–9]. However, one of the them was treated as perianal abscess with no preoperative imaging [9] and another one had a cyst centered in the retrorectal space with extension into the perineal and gluteal areas [8].

Hydatid disease has a variable imaging appearance. There is a standardized WHO classification of the hydatid cyst stages based on the Gharbi ultrasound classification [17, 18]. The hydatid cyst stages are a cyst with a visible cyst wall (stage 1), multiseptated, multivesicular cysts (stage 2), a cyst with a detached floating endocyst membrane (stage 3a), a unilocular cyst with solid or mucinous matrix and internal daughter cysts (stage 3b), a heterogeneous solid cyst with a degenerative internal canalicular structure (stage 4), and a degenerated cyst with heavily calcified wall (stage 5) [17]. It is believed that the first two stages are active, the last two stages are inactive, and stage three is transitional. Treatment decisions are guided by imaging findings and therefore the adaptation of ultrasound-based classification of hydatid cyst into MR and CT imaging has been well described [19–21].

Stojkovic et al. compared the MR and CT imaging findings with ultrasound and concluded that the hydatid cyst matrix is the main defining feature of cyst stage. Also, the “double line sign” is diagnostic for hydatid stage 1. The cyst wall calcifications play some role in defining cyst stage 5 which may be challenging on MRI. Therefore when compared to ultrasound, MRI performs very well with WHO hydatid cyst stages 1, 2, 3, and 4. T2-weighted MR images provide the best sequence to detect cyst contents, that is, septa (stage 2) and daughter cysts (stage 3b) [21]. Singh and Gibikote concluded that T2-weighted MRI is also the best sequence to show the parent capsule and the detached germinal membrane. Both T2-weighted and T1-weighted images should be used to differentiate between the fluid signal intensity of the parent cyst and daughter cysts [22]. The rim sign is described as a low signal intensity rim that is suggestive of hydatid disease rather than other nonparasitic processes. It is more evident on T2-weighted MR images and represents the pericyst and the parasitic membranes. It has been described in hepatic and pulmonary hydatid disease but this sign lacks specificity [6, 23]. CT is superior to ultrasound and MRI in the detection of calcifications and therefore CT is the diagnostic standard for stage 5. In all other stages where the cyst matrix is an important classifying feature, CT performs moderately well compared to ultrasound [21].

## 4. Conclusion

While a tailgut cyst is a rare entity, it should be considered when there is a presacral multilocular mass. Hydatid disease may affect any part of the human body and often has nonspecific imaging findings and serological tests are not always positive. The simultaneous contiguous occurrence of both diagnoses is rare. Attention to the unique imaging features of each cystic lesion facilitates the diagnosis. Clinicians and radiologists should entertain hydatid cysts in their differential diagnoses whenever they encounter cystic lesions especially if the patient is from an endemic area.
